# KaiXinSan-JiaWei ameliorates cognitive dysfunction in APP/PS1 mice by intervening in gut microbiota and its metabolites

**DOI:** 10.3389/fphar.2025.1483883

**Published:** 2025-04-25

**Authors:** Lulu Mana, Fang Chen, Xiaoxia Yuan

**Affiliations:** ^1^ Institute of Traditional Chinese Medicine, Xinjiang Medical University, Urumqi, Xinjiang, China; ^2^ Traditional Chinese Medicine Hospital Affiliated with Xinjiang Medical University, Urumqi, Xinjiang, China

**Keywords:** Kai Xin San-Jia Wei, Alzheimer’s disease, 16S rRNA, SCFAs, microbial-gut brain Axis, intestinal barrier

## Abstract

**Background:**

Alzheimer’s disease (AD) is a degenerative disease of the central nervous system characterized by progressive cognitive impairment and memory loss. Chinese medicine’s therapeutic effect on AD has become a promising treatment option in recent years, and the Chinese herbal compound Kai Xin San-Jia Wei (KXSJW) is one of its representatives. This study employed a comprehensive approach, including 16S rRNA and Gaschromatography-mass spectrometry (GC-MS) analysis, to investigate the therapeutic efficacy and intrinsic mechanism of KXSJW on AD.

**Methods:**

50 3-month-old APP^swe^/PS1^dE9^ transgenic mice were randomly divided into 5 groups (n = 10): model group (M), donepezil group (Don), KXSJW-low dose group (KJW-L), KXSJW- medium dose group (KJW-M), KXSJW-high dose group (KJW-H), Ten 3-month-old C57BL/6 J wild-type mice were randomly selected as the control group (C). The control and model groups were administered saline by gavage, the donepezil group was administered donepezil (0.92 mg/kg/d), and the KXSJW-low/medium/high dose group was administered KXSJW extract (0.9/1.8/3.6 mL/kg/d); each group was treated once daily for 2 months. The study employed the Morris Water Maze (MWM) to evaluate learning and cognitive abilities. Pathological changes in colon tissue were assessed through hematoxylin and eosin (HE) staining. Analysis of gut microbiota was conducted using 16S rRNA sequencing, and gut microbial metabolite (short chain fatty acids, SCFAs) content was detected using GC-MS. Colonic tissue barrier integrity was examined through immunohistochemistry and western blot, while β-amyloid deposition in brain tissue was assessed. ELISA was used to measure serum intestinal peptide hormones (Glucagon, GHRP-Ghrelin).

**Results:**

KXSJW enhanced learning ability and memory, reduced amyloid deposition in the brain tissue of AD model mice. KXSJW was able to restore the balance of intestinal flora and regulate the concentration of intestinal flora metabolites, especially represented by *Firmicutes* and its major metabolite butyric acid. Meanwhile, KXSJW restored the intestinal barrier function and improved the release level of intestinal peptide hormones (Glucagon, GHRP-Ghrelin) in AD model mice. This indicates that KXSJW can improve the intestinal internal environment of AD model mice.

**Conclusion:**

KXSJW may improve the homeostasis of the gut environment in AD, with a focus on the regulation of gut microorganisms and their metabolites, and subsequently improve cognitive impairment in AD. Traditional Chinese Medicine (TCM) has the potential to intervene in AD through multilevel interaction with the brain-gut-axis.

## 1 Background

Alzheimer’s disease (AD) is an age-related neurodegenerative disorder that imposes a dual physiological and psychological burden on the elderly and their families. The disease causes irreversible pathological changes and reduces patients’ ability to self-care while altering their personalities. However, treatments for AD are currently limited although early diagnosis and timely, comprehensive treatment can be of benefit. The gut flora comprises a vast multitude of microorganisms that colonize the gastrointestinal (GI). The maintenance of a healthy gut flora ensures the stability of the intestinal environment. Furthermore, this microflora is involved in the metabolization of an extensive range of active substances, including short chain fatty acids (SCFAs). Additionally, there is evidence to suggest that gut flora may influence brain functions (Liang et al., 2024; Loh et al., 2024). Modifications in the composition of the gut flora result in enhanced permeability and immune activation of the gut barrier (König et al., 2016; Sochocka et al., 2019), which in turn causes systemic inflammation. This may result in the compromise of the blood-brain barrier (BBB) and the promotion of neurological damage, ultimately leading to the development of neurodegenerative diseases, including AD:there is an increasing amount of evidence that suggests a correlation between gut flora and AD (Chandra et al., 2023; Grabrucker et al., 2023; Qu et al., 2023) Modulating the gut microbiota has been considered a promising therapeutic approach; including dietary change (Hoscheidt et al., 2022), exercise (Ngandu et al., 2015) and probiotic therapy (Hsu et al., 2023).

Traditional Chinese medicine (TCM) is recognized for its therapeutic potential in treating AD (Ding et al., 2022; Ma et al., 2023). Additionally, TCM has been found to be effective in correcting the composition of the intestinal flora in AD patients, whether as a single ingredient (Fu et al., 2023; Xu et al., 2023a), or in compound form (Su et al., 2020; Jin et al., 2023). Kai-Xin-San (KXS) is a famous, classic prescription recorded in the ancient Chinese medical text ‘Bei Ji Qian Jin Yao Fang’ by the physician Sun Simiao during the Tang Dynasty. The main components of KXS are Polygalae Radix (*Polygala tenuifolia* Willd.), Ginseng Radix et Rhizoma (*Panax ginseng* C. A. Mey.), Acori Tatarinowii Rhizoma (*Acorus tatarinowii* Schott), and Poria cocos (*Poria cocos* (Schw.) Wolf). In TCM, KXS is primarily used to treat forgetfulness and depression, with the added benefits of nourishing the “heart”, calming the mind and strengthening the will, making it a promising prescription for AD (Luo et al., 2023). Polygala saponins are the main active ingredient in Polygalae Radix. Pharmacological studies have demonstrated that Polygala can inhibit neuronal apoptosis (Li et al., 2023), reduce β-amyloid production (Li et al., 2016), and improve cognitive deficits in APP^swe^/PS1^dE9^ transgenic mice, a well characterized AD animal model (Wang et al., 2021). Ginseng contains various chemical constituents, including saponins, polysaccharides and volatile oils. The main active ingredients are ginsenosides protopanaxatriol Rg1 (Rg1), protopanaxadiol Rb1 (Rb1) and protopanaxadiol Re (Re). Studies have confirmed that ginseng may have therapeutic effects on AD through multiple targets and pathways (Zhang et al., 2023; Park et al., 2024; She et al., 2024). β-Synephrine and α-Synephrine are the main active constituents of Acori Tatarinowii Rhizoma. They can cross the blood-brain barrier (BBB) and are used in the treatment of AD (Venkatesan, 2022; Xu et al., 2023b; Zhang et al., 2024). Poria cocos contains triterpenoids and polysaccharides and has been shown to be a potent acetylcholinesterase (AChE) inhibitor by high-speed counterflow chromatography (HSCCC) (Wu et al., 2023). Additionally, in this study, KXS was supplemented with Epimedium (*Epimedium brevicornum* Maxim.) and schisandra (*Schisandra chinensis* (Turcz.) Baill, based on the results of previous pharmacological studies and the clinical experience of TCM in treating AD. Epimedium is a traditional Yang tonic that can inhibit the progression of AD through pathways such as gut flora and brain energy metabolism (Xie et al., 2022; Liu et al., 2023; Zheng et al., 2023). The active ingredients in schisandra corrected gut microbiota dysbiosis in AD rats (Fu et al., 2023), improved spatial learning and long-term memory function, and attenuated inflammatory damage and oxidative stress in AD model rats (Zhao et al., 2023). Therefore, with the aim of improving therapeutic efficacy, the two herbs mentioned above were added to KXS, resulting in Kai Xin San-Jia Wei (KXSJW).

TCM is typically a decoction of herbs ingested orally with absorption in the gastrointestinal tract, where the herbs come into contact with the intestinal flora which may affect their therapeutic effects. It follows that it is important to investigate the effects of herbal preparations on the gut flora. The aim of this study was to investigate whether KXSJW interfered with intestinal flora pathways and their metabolites in treatment of AD. This also aims to assist with the more general exploration of the efficacy of TCM. The study evaluated the efficacy of KXSJW on learning and memory abilities of APP^swe^/PS1^dE9^ mice. The effects of KXSJW on the gut microbiota and their metabolites were evaluated by analyzing changes in gut flora and short chain fatty acids (SCFAs) in the feces of AD model mice. In addition, the effects of KXSJW on the intestinal barrier were studied in the APP^swe^/PS1^dE9^ mice model of intestinal permeability.

In summary, this study was performed to establish a KXSJW-treated AD animal model and to elucidate the effects of KXSJW in the treatment of AD. The aim is to provide new insights into the potential therapeutic role of KXSJW in AD, highlighting its multifaceted effects on learning cognitive function, gut microbiota and their metabolites, and hormonal homeostasis.

## 2 Materials

### 2.1 Drugs and reagents

The KXSJW was composed of Polygalae Radix (*Polygala tenuifolia* Willd.), (Yuanzhi) 15g, Ginseng Radix et Rhizoma (*Panax ginseng* C. A. Mey.), (Renshen) 9g, Acori Tatarinowii Rhizoma (*Acorus tatarinowii* Schott) (Shichangpu) 12g, Poria cocos (*Poria cocos* (Schw.) Wolf (Fuling) 15g, Epimedium (*Epimedium brevicornum* Maxim.), (Yinyanghuo) 24g and schisandra (*Schisandra chinensis* (Turcz.) Baill (Wuweizi) 15g. All herbal components were purchased from Xinjiang Baicaotang Medicine Chain Store Distribution Co., Ltd. (Xinjiang, China) and were validated by Professor Ping Sheng in Xinjiang Medicine University, according to the 2020 edition of the Chinese Pharmacopoeia.

Hydrochloric acid donepezil tablets (5 mg), acting as a positive control in this study, were obtained from Eisai (China) Pharmaceutical Co., Ltd. Methyl tert-butyl ether (CNW Technologies, CAS No. 1634-04-4, HPLC grade); Sulfuric acid (Sinopharm, CAS 7664-93-9, AR); 2-Methylvaleric acid (CAS 97-61-0, ≥99.5), Acetic acid (CAS 64-19-7, ≥99.5), Propionic acid (CAS 79-09-4, ≥99.5), Isobutyric acid (CAS 79-31-2, ≥99.5), Butyric acid (CAS 107-92-6, ≥99.5), Isovaleric acid (CAS 503-74-2, ≥99.5), Valeric acid (CAS 109-52-4, ≥99.5), Hexanoic acid (CAS 142-62-1, ≥99.5), Heptanoic acid (CAS 111-14-8, ≥99.5), Octanoic acid (CAS 124-07-2, ≥99.5), Nonanoic acid (CAS 112-05-0, ≥99.5) and Decanoic acid (CAS 334-48-5, ≥99.5) were all obtained from Dr. Ehrenstorfer. Methanol (Fuchen, CAS 67-56-1, AR); Mouse Glucagon Elisa Kit (JL20654) and GHRP-Ghrelin Elisa Kit (JL20543) were obtained from Shanghai Jianglai Biotechnology Co., Ltd. Antibodies used: anti-Aβ_1-42_ (ab201060, Abcam), anti-occludin (GB111401-100, Servicebio), anti-ZO-1 (GB111402-100, Servicebio), β-actin (LF201, Epizyme). 3,3′-Diaminobenzidine tetrahydrochloride (DAB) (ZLI-9019) was obtained from Beijing ZhongShanJinqiao Biotechnology Co., Ltd. Bicinchoninic Acid Assay (BCA) kit (PC101) and Polyvinylidene difluoride (PVDF) membranes (WJ003; WJ002) were obtained from Shanghai Epizyme Biomedical Technology Co., Ltd. Enhanced chemiluminescence (ECL) kit (BL520A) was obtained from Labgic Technology Co., Ltd. (Biosharp). E.Z.N.A.^®^ Stool DNA Kit (Omega Bio-Tek, OMEGD4015-02) was obtained from Merck.

### 2.2 Animals

Specific pathogen-free (SPF)-grade male APP^swe^/PS1^dE9^ double-transgenic mice and C57BL/6 J mice weighing 22–25 g were obtained from JiangSu HuaChuangXinNuo Bioengineering Co., Ltd. [SCYK(Su) 2020-0009]. All animals were housed in the SPF grade animal laboratories at the Animal Experimentation Centre of Xinjiang Medical University [SCYK(Xin) 2018-0003]. They were maintained under standard laboratory conditions (22°C–25°C, a 12-hour light/dark cycle) with food and water *ad libitum*. All animal experiments were performed according to European Community guidelines (EEC Directive of 1986; 86/609/EEC) and were approved by the Ethics Committee of the Animal Experimentation Centre of Xinjiang Medical University (IACUC-20200331-137).

### 2.3 Instruments

Equipment included: Morris water maze (MWM) video analysis system (ZS-001, Zhongshi Dichuang Technology), Gas Chromatograph (GC-2030, Shimadzu); Mass Spectrometer (QP2020 NX, Shimadzu); Columns (HP-FFAP, 30 m × 250 μm × 0.25μm, Agilent Technologies); Centrifuge (Heraeus Fresco17, Thermo Fisher Scientific); Ultra-low temperature refrigerator (Forma 900 series, Thermo Fisher Scientific); Analytical Balance (BSA124S-CW, Sartorius); Grinder (JXFSTPRP-24OMIT GAP HERE, Shanghai Jingxin); Ultrasonograph (PS-60AL, Shenzhen leaderbang); Vacuum drying oven (DZF-6096, Shanghai Yiheng); Dual Plate Vertical Electrophoresis (DYCZ-24DH, Beijing LIUYI); and Paraffin microtome (Thermo Fisher, United States).

## 3 Methods

### 3.1 Preparation of herbal extracts

KXSJW preparation was carried out based on previous reports (Yu et al., 2021). The herbs were mixed and boiled in eight volumes of double-distilled water for 30 min, the extracts were poured off, water was added to the herbs and boiled again, and the extracts from the two decoctions were combined. The resulting extracts constituted the KXSJW preparation. Each Gram of KXSJW extract was equivalent to 5.17 g of raw herbs.

### 3.2 Animal grouping and drug administration

Fifty 3-month-old APP^swe^/PS1^dE9^ transgenic mice were randomly divided into 5 groups according to a random number table (n = 10 each group): the model group (M), the donepezil group (Don), the KXSJW-low dose group (KJW-L), the KXSJW-medium dose group (KJW-M), and the KXSJW-high dose group (KJW-H). A 6^th^ group, which included 10 3-month-old C57BL/6 J wild-type mice, chosen as the control group (C). The control and model groups were administered saline by gavage, the donepezil group was administered donepezil (0.92 mg/kg/d), and the KXSJW-low/medium/high dose group was administered KXSJW extract (0.9/1.8/3.6 mL/kg/d). Each group was treated once daily for 2 months and all doses were administered in a volume of 0.1 mL/10g body weight.

### 3.3 Morris water maze (MWM)experiment

Spatial learning and memory were assessed with MWM testing, a combination of a localization navigation test and a spatial exploration experiment. Briefly, a 1.2 m diameter pool was divided into four quadrants, each labelled with a different symbol. The target quadrant was designated as the first quadrant with the escape platform fixed 1 cm below the water surface. In the localization navigation test, mice in each group were placed in the water in quadrant order and the time taken for the mice to reach the platform within 90 s was recorded. If the mice found the platform and remained there for ≥5 s, they were considered to succeed (i.e., escape latency). If mice failed to find the platform in <90 s, they were led to the platform and allowed to remain on the platform for 20 s before returning to the cage. Each mice was trained 4 times/d for 5 days. On day 6, the spatial exploration experiment was performed, the experimental platform was removed, and the number of times the mice entered and crossed the platform in 90s was recorded to assess the learning and memory abilities of the mice.

### 3.4 Sample collection

Mice were anesthetized with 10% sodium pentobarbital (35 mg/kg) body weight. Blood samples, approximately 1 mL, were taken from mice fasted overnight by cardiac puncture 48h after completion of the MWM. The left ventricle was perfused with pre-cooled 4°C saline, and the right atrium was opened with surgical scissors. Perfusion was stopped when the mice liver turned white. The mice brain tissue was removed and the left and right hemispheres were separated. The left hemisphere was fixed with 4% paraformaldehyde. The right hemisphere was rinsed with saline and rapidly placed in liquid nitrogen before being transferred to a −80°C refrigerator, rectal tissue was extracted and preserved using the same method. At least five fecal pellets were collected from the rectum of each mice, placed in sterile freezer tubes and stored at −80°C. Blood was centrifuged at 3,000 rpm for 15 min, serum was separated and stored at −80°C for testing.

### 3.5 Hematoxylin-eosin (HE) staining and immunohistochemical analysis

Fixed mice rectal tissue blocks were embedded in paraffin and 5 μm sections were cut using a microtome. Sections were dewaxed in xylene, hydrated in ethanol (15 min each), rinsed in distilled water (2 min), immersed in hematoxylin stain (5 min), rinsed in tap water (1 min), treated with differentiation solution (2 s), rinsed in tap water (30 s), immersed in eosin stain (1 min), rinsed in tap water, dehydrated, sealed with neutral gum and then viewed under a microscope.

The brain and rectal tissues used for immunohistochemistry were embedded in paraffin and cut into coronal sections of 5 μm thickness. Paraffin sections were deparaffinized and rehydrated after heating at 56°C for 60 min. After microwave repair of antigens, they were blocked with 3% H_2_O_2_ for 30 min to quench endogenous peroxidase activity. Subsequently, the sections were allowed to incubate in goat serum for 30 min at room temperature (RT) and then incubated with primary anti-Aβ_1-42_ (1:1200) antibody, anti-occludin (1:1000) antibody and anti-ZO-1 (1:500) antibody at 4°C overnight. The following day, the sections were incubated with the appropriate secondary antibodies for 30 min at RT. The sections were then rinsed with phosphate buffered saline (PBS), stained with DAB for 6 min and counterstained with hematoxylin. Finally, the sections were examined under a light microscope and representative images were analyzed using ImageJ image analysis software.

### 3.6 Western blotting analysis

Mice rectal tissue was removed from liquid nitrogen, thawed and treated with Radioimmunoprecipitation assay buffer (RIPA) tissue lysis buffer containing protease and phosphatase inhibitors, and the protein concentration in the lysate supernatant was determined using a BCA kit. Equal amounts of sample proteins were prepared for 5%–15% SDS-PAGE analysis. After electrophoresis, proteins were transferred to PVDFmembranes, which were then immersed in 5% skimmed milk for 1h at RT. Primary antibodies anti-occludin (1:1000), ZO-1 (1:1500) and β-actin (1:10,000) were added to detect the target proteins. The PVDF membranes were incubated overnight at 4°C. The next day, after three washes with Tris Buffered Saline with Tween 20 (TBST) the membranes were incubated with the appropriate secondary antibody for 1–2 h at RT. Membranes were visualized using an ECL kitand captured using automated chemiluminescence imaging (E-blot Touch Viewer, e-BLOT). Protein bands were analyzed with ImageJ.

### 3.7 Enzyme linked immunosorbent assay (ELISA) analysis

Mice serum samples were removed from the refrigerator and equilibrated to RT. Following the instructions, standard wells, containing 50 μL of different concentrations of standards, blank wells, containing 50 μL of sample dilution, and sample wells (50 μL of samples to be tested) were set up. 100µL of horseradish peroxidase (HRP) conjugated detection antibody was added to each well, then sealed and incubated at RT for 1 h. The liquid was then discarded, and the samples washed 5 times. Substrate was added to each well, incubated for 15min and termination solution was added. The Optical density (OD) value of each well was measured at 450 nm.

### 3.8 16S rRNA gene sequencing analysis

#### 3.8.1 Fecal sample microbiome total DNA extraction and PCR amplification

Mice were fasted for 12h after completion of the MWM experiment. Fresh fecal samples were collected into sterile containers and stored at −80°C. Samples were transported to Shanghai Biotree BIOTECH Co., Ltd. for 16S rDNA sequencing to analyze the gut microbiota. Total microbiota DNA was extracted from the fecal samples using the E.Z.N.A.^®^ Stool DNA Kit. The quality of DNA extraction was detected by agarose gel electrophoresis, and the DNA was quantified by UV spectrophotometer.

For PCR amplification, primers with specific barcodes for each sample and sequencing universal primers were tagged at the 5′ends (Table 1).

**TABLE 1:** PCR amplification and 16S rDNA sequencing.

Region	Primers
V3-V4	341F (5′-CCTACGGGNGGCWGCAG-3′)
	805R (5′-GACTACHVGGGTATCTAATCC-3′)
Archae	F (5′-GYGCASCAGKCGMGAAW-3′)
	R (5′-GGACTACHVGGGTWTCTAAT-3′)
V4	515F (5′-GTGYCAGCMGCCGCGGTAA-3′)
	806R (5′- GGACTACHVGGGTWTCTAAT-3′)
V4-V5	F (5′-GTGCCAGCMGCCGCGG-3′)
	R (5′-CCGTCAATTCMTTTRAGTTT-3′)

The sequences were amplified using the primers in Table 1 and the PCR products were confirmed by 2% agarose gel electrophoresis. Ultrapure water was used throughout DNA extraction as a negative control, and PCR products were purified by AMPure XT beads (Beckman Coulter Genomics, Danvers, MA, United states) and quantified by Qubit (Invitrogen, United states). Amplicon pools were used for sequencing, and the size and number of amplicon libraries were evaluated on Agilent 2,100 Bioanalyzer (Agilent, United states) and Illumina (Kapa Biosciences, Woburn, MA, United states) library quantification kits, respectively. Libraries were sequenced on the NovaSeq PE250 platform.

#### 3.8.2 bioinformatic analysis

Samples were sequenced on the Illumina NovaSeq platform. Barcodes were used to assign paired-end sequences to samples, and barcode and primer sequences introduced during library construction were removed. Matching end reads were merged using Fast Length Adjustment of SHort reads (FLASH). Raw reads were quality-filtered for high quality clean tags using fqtrim (v0.94). Chimeric sequences were filtered using VSEARCH (v2.3.4). Feature tables and feature sequences were obtained using DADA2. Diversity and diversity were calculated by normalizing to the same random sequence. Feature abundance was then normalized using the relative abundance of each sample according to the SILVA (release 132) classifier. Alpha diversity was used to analyze the complexity of sample species diversity using five metrics including Chao1, observed species, goods coverage, Shannon, and Simpson. Beta diversity was calculated using QIIME2. Sequence comparison was performed using BLAST and each representative sequence was annotated with character sequences using the SILVA database.

### 3.9 Short chain fatty acids (SCFAs) analysis

#### 3.9.1 Sample pre-processing

The sample (6 aliquots) was transferred to Eppendorf (EP) tubes, extracted with H_2_O, and vortex mixed for 10 s. It was homogenized in a ball mill for 4 min at 40 Hz, then ultrasonicated for 5 min; repeated 3 times. After centrifugation for 20 min, at 5,000 rpm, at 4°C, 0.8 mL of the supernatant was transferred to a fresh EP tube, 0.1 mL 50%H_2_SO_4_ was added with 0.8 mL extraction solution (25 mg/L stock in methyl tert-butyl ether) as internal standard. After vortex mixing for 10s, sonication for 10 min, centrifugation for 15 min, at 10,000 rpm, at 4°C it was stored at −20°C for 30 min. The supernatant was transferred to a fresh glass vial to be analyzed by gas chromatography-mass spectrometry (GC-MS).

#### 3.9.2 GC-MS detection and analysis

1 µL of sample was injected in a 5:1 separation mode with helium as the carrier gas, a front inlet septum purge flow of 3 mL min^-1^ and column flow of 1.2 mL min^−1^. Oven temperature was ramped: 50°C held for 1 min; raised to 150°C at a rate of 50°C min^−1^, held for 1 min; raised to 170°C at a rate of 10°C min^−1^, held on 1 min; raised to 225°C at a rate of 20°C min^−1^, held for 1min; raised to 240°C at a rate of 40°C min^−1,^ held on 1 min. The temperatures of the front injection, the transfer line, the ion source and the quad were 240 °C, 240 °C, 200 °C and 150°C, respectively. The electron energy was −70eV. The mass spectrometry data were acquired in Scan/SIM mode with an m/z range of 33–150 after a solvent delay of 3.75 min.

#### 3.9.3 Standard curve establishment and Sample quantification

$$\text{Formula}:C_{\left(\text{CON}\right)}=\frac{C_{s}\times v_{1}\times v_{3}}{M\times v_{2}}\times 1000$$

where C_(CON)_: represented content of target compounds in the sample (μg/g); Cs: the concentration of target compounds in the extraction solution (mg/L); V_1_: the volume of extract solution added (mL); V_2_: the volume of supernatant liquid removed from de-ionized water (mL); V_3_: the volume of de-ionized water added (mL); and M: the weighed total volume (mg).

### 3.10 Statistical analysis

Statistical analyses were performed using SPSS 25.0, graphs were generated using GraphPad Prism 6.0. Data were expressed as mean ± standard deviation (X±SD). Homogeneity of variance was compared by one-way analysis of variance *(ANOVA)* and *Tukey’s post hoc* test when the mean of 3 or more groups was a continuous normally distributed variable. The *Kruskal–Wallis test* was used to compare heterogeneity of the means and variance of non-normally distributed variables in 3 or more groups. *p* < 0.05, was taken to indicate statistical significance. Beta diversity analysis of intestinal flora was performed using principal component analysis (PCA), and Principal coordinates analysis (PCoA).

## 4 Result

### 4.1 KXSJW arrested cognitive deficit and attenuated β-amyloid deposition in brain tissue ofin APP^swe^/PS1^dE9^ AD model mice

**FIGURE 1:**
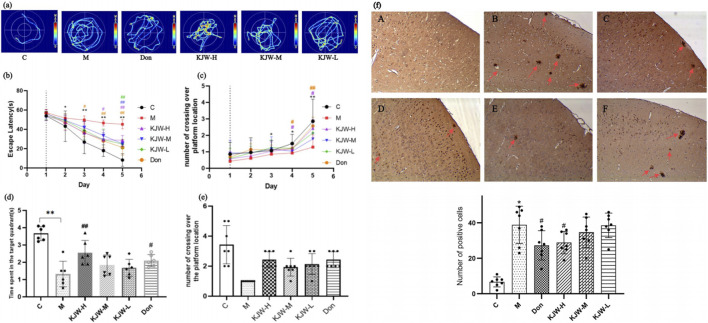
Morris water maze experiment **(a–e)**: effects of different treatments. **(a)** Swimming trajectory maps of mice in localization navigation experiment. **(b)** Latency of mice in the localization navigation test. **(c)** The number of times the mice crossed the platform during the localization navigation test. **(d)** Length of time (mean ± SD) mice explored the target quadrant in the spatial exploration test. **(e)** Number of times (mean ± SD) the mice crossed over the platform in the space exploration test. Immunohistochemistry analysis of β-amyloid deposition in different groups of mice (F) **(a)**, control group (C); **(b)**, model group (M); **(c)**, donepezil group (Don); **(d)**, KXSJW-high dose group (KJW-H); E, KJW-middle dose group (KJW-M); **(f)**, KJW-low dose group (KJW-L). The positive cells are signified by red arrows. *p < 0.05; **p < 0.01, compared to the control group, ^*#*^ *p* < 0.05, ^*##*^ *p* < 0.01, compared to the model group.

The experiment conducted during the first 5 days was designed to test learning and memory abilities of mice through positioning navigation. On the 6th day, a spatial exploration test was conducted to evaluate memory retention. The Figure 1 shows that the latency period was prolonged in the model group compared to the control group from day 2 of the orientation trial (*P* < 0.05). On day 5, the latency period showed a significant prolongation (*P* < 0.05), indicating that the mice had decreased learning memory function. In contrast, the latency period and the number of platform crossings of the mice in the KJW-H group declined from the 3rd day becoming significant by day 4 compared to the model group. The spatial exploration experiment results indicated that the model group of mice exhibited a significant decrease in memory retention, as evidenced by a lower number of traversing platforms and lower dwell time in the target quadrant (*P <* 0.05). However, each KJW dose group demonstrated varying degrees of improvement in memory retention of AD mice (*P <* 0.05).

Immunohistochemistry was performed to assess the extent of β-amyloid deposition in mice brain tissue. As shown in Figure 1f, a significant increase in β-amyloid deposition was observed in the brain tissues of mice in the model group, compared to controls (*P* < 0.05). KJW-H significantly reduced β-amyloid deposition in the brain tissues of APP^swe^/PS1^dE9^ AD model mice.

### 4.2 KXSJW regulates gut microbiota composition in APP^swe^/PS1^dE9^ AD model mice

**FIGURE 2:**
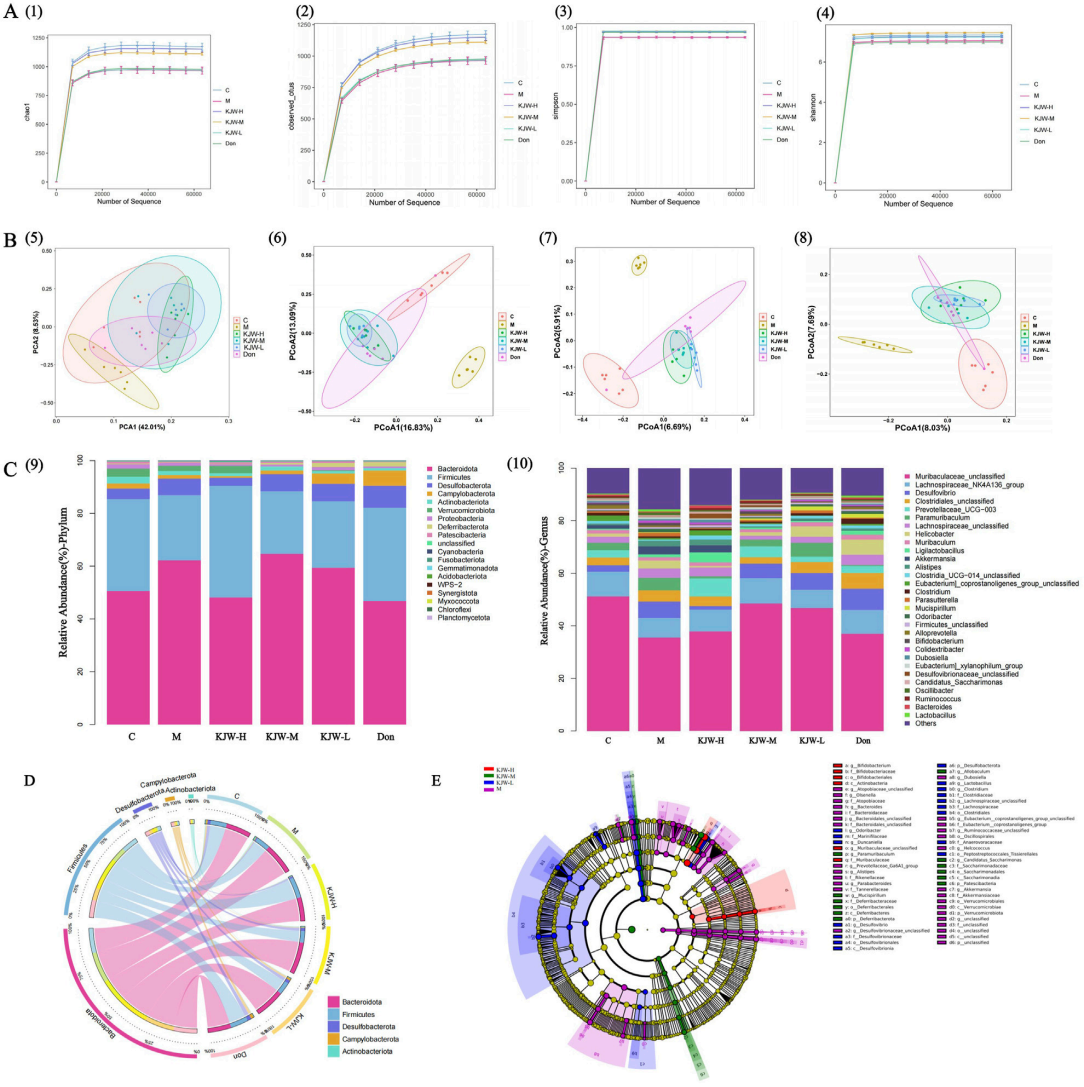
16S rRNA gene sequence analysis: effect of KXSJW on intestinal flora of APP^swe^/PS1^dE9^ AD model mice A (1): Chao1. **(A)** (2): observed_species, *Y*-axis: observed species operational taxonomic units (OTUs). Simpson A (3) and Shannon A (4) were assessed for the comparison of alpha diversity. **(B)** (5): PCA of the fecal microbial community. PCoA of the fecal microbial community based on Bray-Curtis dissimilarity distance function B (6) and Jaccard similarity coefficient B (7). Unweighted unifrac distance matrices analysis B (8), the percentages on the horizontal and vertical coordinates indicate the degree to which PCoA1 and PCoA2 explain the differences between the samples. **(C)** (9): Relative abundance of the top 19 members of the microbial community at phylum level. **(C)** (10): Relative abundance of the top 30 members of the microbial community at genus level. **(D)** The Circos graph illustrates the distribution of the abundance of the 5 most populous colonies. **(E)** Linear Discriminant Analysis (LDA) Effect Size (LEfSe) Analysis comparing the model group, and KJW-H, KJW-M, KJW-L groups.

The alpha diversity of the gut microbiota in each group of mice was assessed using the Chao1, observed species, Shannon and Simpson indices. Figures 2A(1, 2) show that the model group of mice had reduced Chao1 and observed_species indices compared to the control group. The KJW-H and KJW-M groups had increased Chao1 and observed_species indices compared to the model group, although the differences did not achieve statistical significance. This suggests that KXSJW had the potential to affect the gut microbial abundance in mice. Shannon and Simpson indices were used to evaluate microbial community diversity. Figure 2A(3, 4) show a decreasing trend in the intestinal microbial diversity of mice in the model group compared to the control group. KXSJW improved the trend of Shannon and Simpson indices in APP^swe^/PS1^dE9^ mice although this did not achieve statistical significance. This suggests that KXSJW may improve the intestinal microbial diversity of AD mice. Principal Component Analysis (PCA) and Principal Coordinate Analysis (PCoA) were used to compare beta diversity between groups. The results, shown in Figure 2B (5), suggest a difference in the gut microbiome between model and control group mice. Similarly, PCoA analysis based on Bray-Curtis dissimilarity distance function, Jaccard similarity coefficient and unweighted unifrac distance matrices shown in Figure 2B(6–8) indicated differences in microbial composition between the groups.

The composition of the gut microbiota of each group was further analyzed at the level of phyla and genera, and the results are shown in Figure 2C (9,10). At the level of phyla, the most important groups are *Bacteroidota, Firmicutes, Desulfobacterota, Campylobacterota, Actinobacteriota*, *Verrucomicrobiota* and *Proteobacteria*. The relative abundance of *Bacteroidota, Desulfobacterota* and *Proteobacteria* increased in the model group and *Firmicutes, Actinobacteriota, Verrucomicrobiota* showed a decreasing trend. KXSJW improved the abundance of *Bacteroidota* and *Firmicutes*. At the genus level, the abundance of *Muribaculaceae_unclassified* and *Lachnospiraceae_NK4A136_group* showed a decreasing trend in the model group. KXSJW increased their abundance in the gut microbiota of APP^swe^/PS1^dE9^ mice. The same results are presented in a more visual way in Figure 2D.

The LEfSe Analysis was employed to compare the model group with each KXSJW dose group, aiming to identify species exhibiting significant differences in abundance across different groups, as shown in Figure 2E. Higher LDA scores were observed for *g_Bifidobacterium*, *f_Bifidobacteriaceae*, *o_Bifidobacteriales*, *c_Actinobacteria* and *f_Muribaculaceae* using LEfSe.

### 4.3 KXSJW regulates intestinal flora metabolites in APP^swe^/PS1^dE9^ AD model mice

**FIGURE 3:**
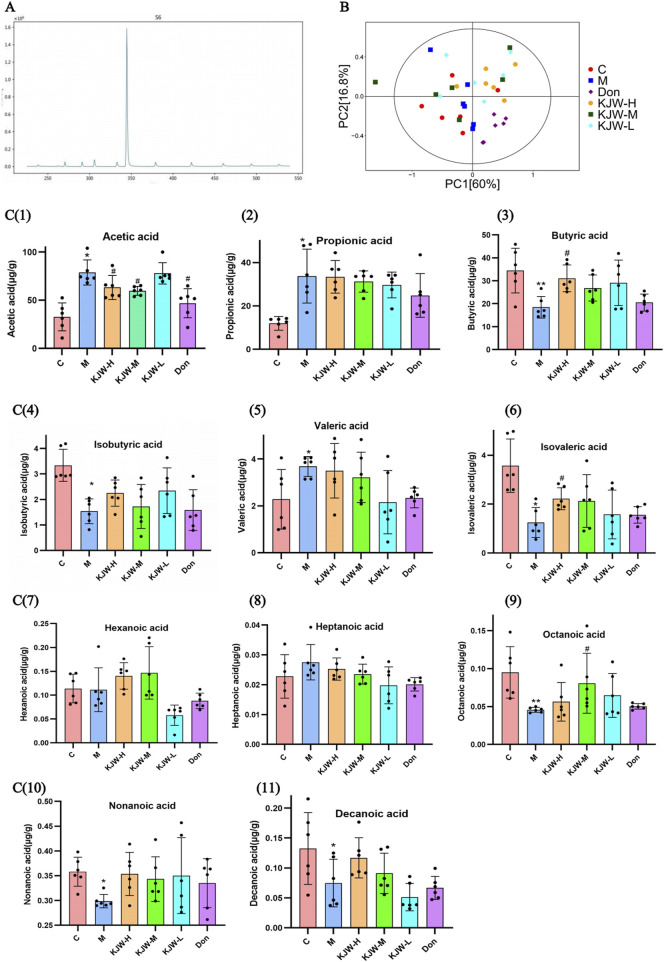
GC-MS Selected Ion Monitoring (SIM) Ion Flow Diagram of Target SCFAs Standards **(A)** and GC-MS target detection of changes in SCFAs in feces of mice in each group **(B, C)**. **(B)** principal component analysis (PCA) analysis. (C, 1-11) The SCFAs levels in response to KXSJW-treatment in APP^swe^/PS1^dE9^ AD model mice.

The metabolites of gut microbiota (SCFAs) were detected by GC-MS, and the GC-MS SIM Ion flow diagram of target compound standards is shown in Figure 3A. The results of PCA analysis are shown in Figure 3B, indicating differences in the composition of fecal SCFAs in different groups of mice, the horizontal and vertical coordinates Principal Component 1 (PC1) and Principal Component 2 (PC2) represent the scores of the first and second principal components, respectively. The scatter plots represent individual samples and the colors and shapes of the scatters indicate distinct groupings. The samples are situated within Hotelling’s T-square ellipse, which is indicative of a 95% confidence interval. The effect of KXSJW-treatment on SCFAs in APP^swe^/PS1^dE9^ AD model mice is shown in Figure 3 (C, 1-11). Acetic acid, propionic acid and valeric acid showed a significant increase in the model group compared to the control group (*P <* 0.05), while KJW-H and KJW-M significantly increased acetic acid compared to the model group (*P <* 0.05). Butyric acid, isobutyric acid, isovaleric acid, octanoic acid, nonanoic acid, and decanoic acid were significantly decreased in the model group compared to the control group (*P <* 0.05). KJW-H caused a significant increase in butyric acid and isovaleric acid compared to the model group (*P <* 0.05), while KJW-M caused a significant increase in octanoic acid compared to the model group (*P <* 0.05). The results suggest that KXSJW may have a regulatory effect on the metabolites of intestinal flora in APP^swe^/PS1^dE9^ AD model mice, but dosage may be important.

### 4.4 KXSJW exerts a protective effect on the intestinal mucosal barrier in APP^swe^/PS1^dE9^ AD model mice

**FIGURE 4:**
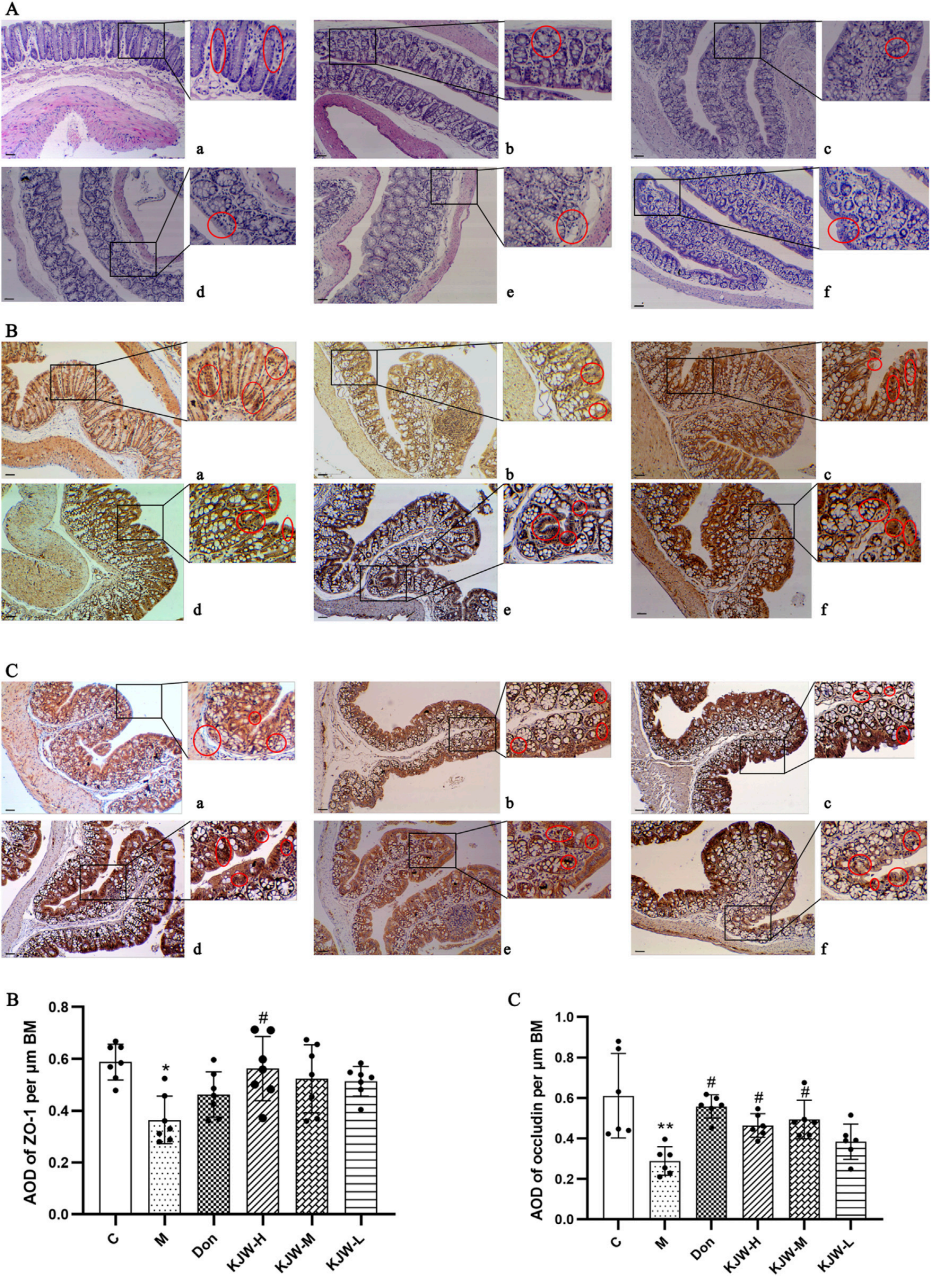
The Effect of KXSJW on the intestinal barrier in APP^swe^/PS1^dE9^ AD model mice. **(A)** HE staining was used to assess the colonic morphology in different groups of mice. Immunohistochemical analysis (mean ± SD) showing expression of Zonula Occludens-1 (ZO-1) **(B)** and occludin **(C)** in different groups. **(a)** Control group (C), **(b)** model group (M), **(c)** KXSJW-high dose group (KJW-H), **(d)** KJW-middle dose group (KJW-M), **(e)** KJW-low dose group (KJW-L), **(f)** donepezil group (Don). Areas of positive staining are marked with red circles. *p < 0.05; **p < 0.01, compared to the control group, ^*#*^ *p* < 0.05, ^*##*^ *p* < 0.01, compared to the model group.

Histopathological changes in the colon were examined to determine the effect of KXSJW on intestinal pathology. Figures 4A(a–f) shows that the colons of APP^swe^/PS1^dE9^ mice exhibited slight mucosal atrophy and villous destruction, while the control group did not show any damage to the villi or epithelial destruction. However, treatment with KXSJW had a beneficial effect in reducing colonic lesions in APP^swe^/PS1^dE9^ mice. Immunohistochemical assay results shown in (Figures 4B, C) revealed a significant reduction in the protein expression of ZO-1 (B) and occludin (C) in the colonic tissues of the model group compared to the control group (*P <* 0.05). KJW-H significantly increased the protein expression of ZO-1 in the colonic tissues of APP^swe^/PS1^dE9^ mice Figures 4B(d), and KJW-H and KJW-M significantly increased the protein expression of occludin in the colonic tissues of APP^swe^/PS1^dE9^ mice Figures 4C(d, e) (*P <* 0.05).

**FIGURE 5:**
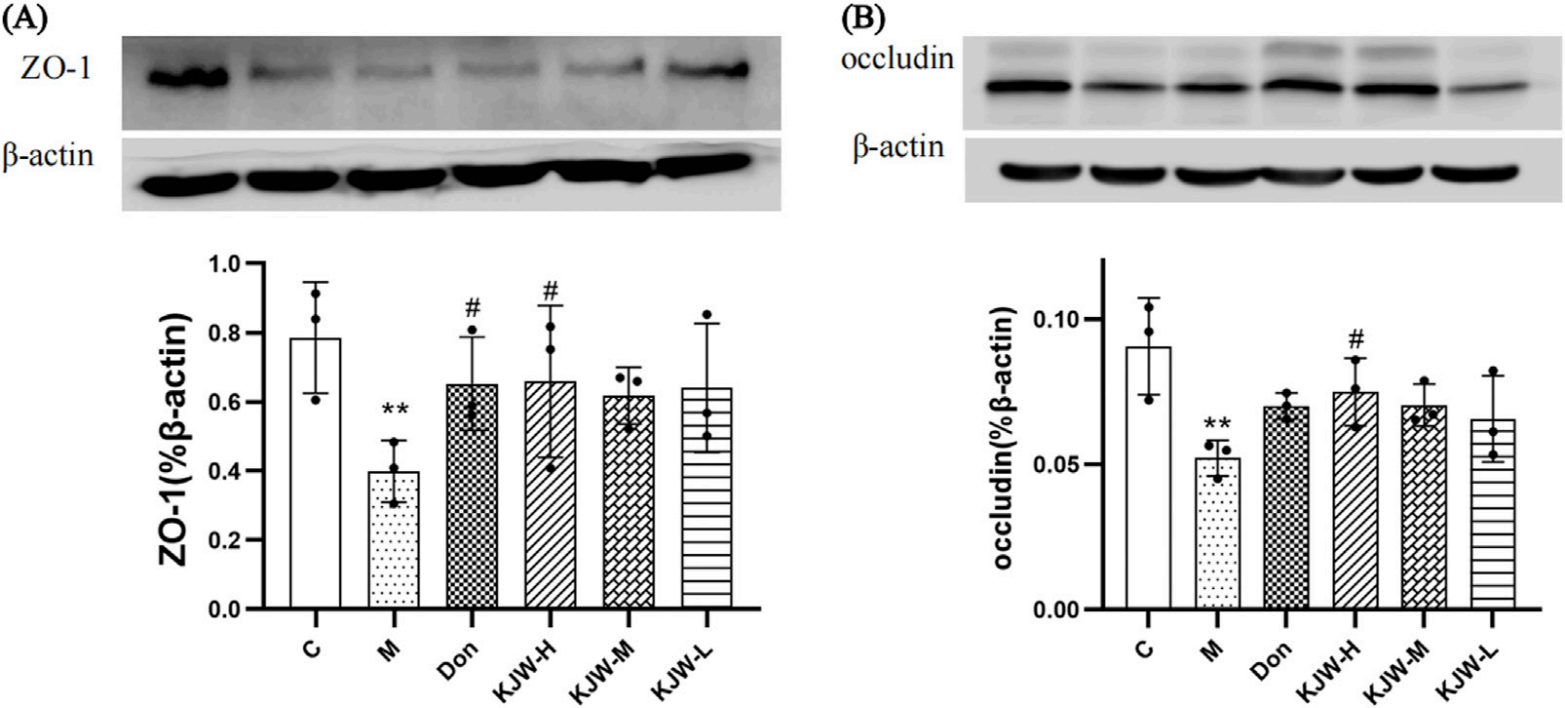
Western blot analysis (mean ± SD) of ZO-1 **(A)** and occludin **(B)** expression in different groups. (C) Control group, (M) model group (KJW-H) KXSJW-high dose group, (KJW-M) KJW-middle dose group, (KJW-L) KJW-low dose group, (Don) donepezil group. *p < 0.05; **p < 0.01, compared to the control group, ^*#*^ *p* < 0.05, ^*##*^ *p* < 0.01, compared to the model group.

Western blot analysis was employed to further evaluate the changes in protein expression of ZO-1 and occludin in the colonic tissues of each group. Figure 5 illustrates that the colonic tissue protein expression of ZO-1 (A) and occludin (B) in the model group mice was significantly decreased (*P <* 0.05). KXS-H significantly increased the colonic tissue protein expression of ZO-1 and occludin (*P <* 0.05) in the APP^swe^/PS1^dE9^ mice, indicating that KXSJW may have a protective effect on the intestinal barrier of APP^swe^/PS1^dE9^ mice.

### 4.5 KXSJW regulates serum GHRP-Ghrelin and glucagon in APP^swe^/PS1^dE9^ AD mice

**FIGURE 6:**
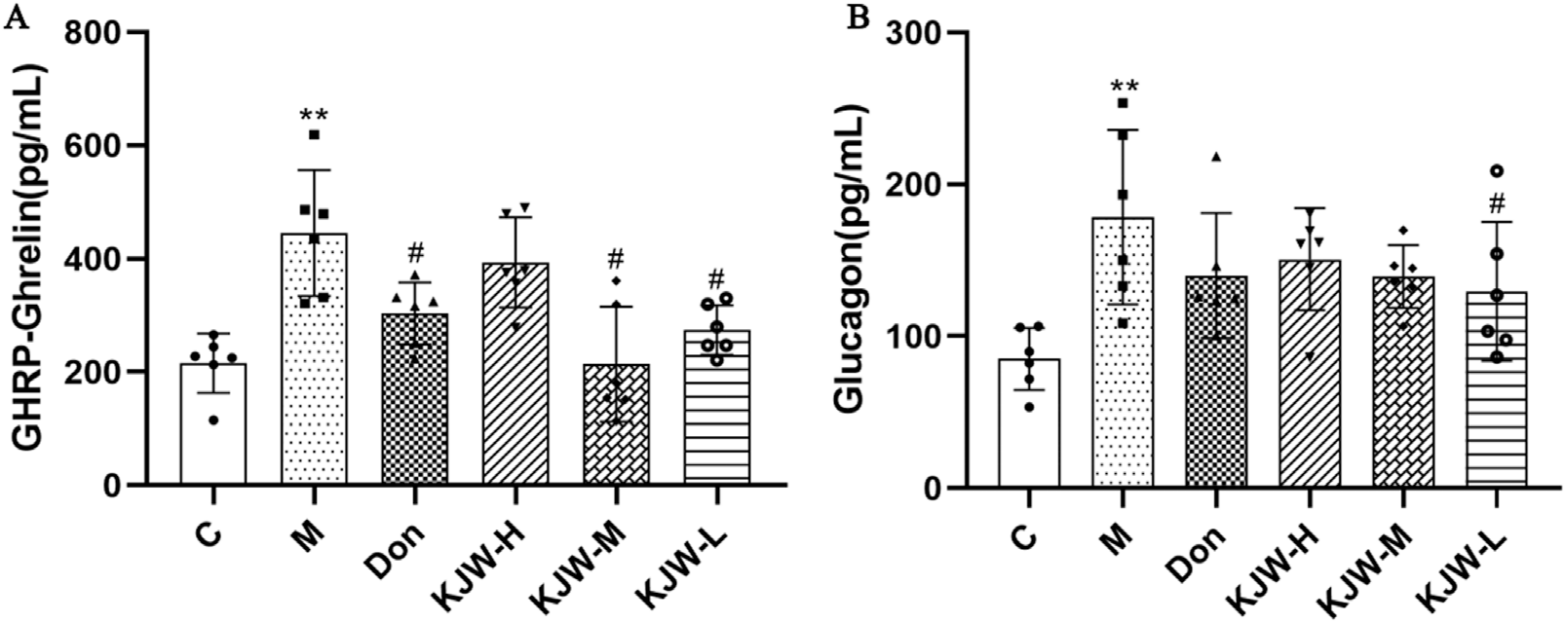
ELISA analysis (mean ± SD) of serum GHRP-Ghrelin **(A)** and Glucagon **(B)** release in different groups. (C) Control group, (M) model group (KJW-H) KXSJW-high dose group, (KJW-M) KJW-middle dose group, (KJW-L) KJW-low dose group, (Don) donepezil group. *p < 0.05; **p < 0.01, compared to the control group, ^*#*^ *p* < 0.05, ^*##*^ *p* < 0.01, compared to the model group.

Serum glucagon and GHRP-Ghrelin release were measured by ELISA. Figure 6 shows that serum Glucagon and GHRP-Ghrelin were significantly elevated in the model group, compared to the control group (*P <* 0.05), indicating metabolic dysregulation. KJW-M and KJW-L significantly decreased serum GHRP-Ghrelin, and KJW-L significantly decreased serum Glucagon release in APP^swe^/PS1^dE9^ AD mice, suggesting that KXSJW can regulate metabolic disorder in AD mice, but dosage may be important.

## 5 Discussion

AD is the most the most common cause of dementia accounting for approximately two-thirds of all dementia cases and affecting more than 35 million people world-wide.

Existing studies of pathological mechanisms and clinical treatments for AD have begun to focus on the crosstalk between gut flora and homeostasis in the brain. A significant difference has been revealed in the abundance, diversity, and metabolite content of the gut flora composition in AD patients compared to that of the normal population (Ma et al., 2024). Medications to rebuild the gut flora have been reported to have a significant therapeutic effect in improving cognition in AD patients (Fei et al., 2023). These findings suggest that the microbial-gut-brain axis is a potentially important pathway that influences the onset and development of AD.

The intestinal mucosal barrier is a highly selective and functional barrier system that maintains the stability of the internal environment of GI (Che et al., 2022). The intestinal mucosal barrier can be classified as a mechanical barrier, a biological barrier, a chemical barrier, and an immune barrier. As previously stated, damage to the intestinal mucosal barrier can affect the central nervous system (CNS) through the microbial-gut-brain axis. The extensive gut flora and its metabolites (i.e., SCFAs) are considered to constitute the biological barrier of the GI. The intestinal mucosal epithelium and the tight junctions between the cells comprise the mechanical barrier. The various types of hormones produced by the enteroendocrine cells (EECs), notably GHRP-Ghrelin, leptin, and GLP-1, are considered to act as the chemical barrier. Furthermore, the metabolites derived from the intestinal microbiota have been demonstrated to modulate the intestinal hormone secretion of the EECs (Ma et al., 2023).

Animal models of AD have been widely used to understand pathogenesis and for pre-clinical testing of novel therapies. Transgenic mouse models have been most commonly used and APP^swe^/PS1^dE9^ mice have been studied with progressive deposition of beta-amyloid plaques, neurofibrillary involvement, parenchymal and vascular deposits and validated experimental measurements (Alhabeef et al., 2021).

In this study, we found that in addition to impaired learning and memory, APP^swe^/PS1^dE9^ mice also had impaired gut barrier function and gut microbiota. Our study employed a 16S rRNA assay to investigate the potential mechanisms of the role of KXSJW in the pathogenesis of AD. Significant differences in gut bacteria composition and their metabolites between untreated and KXSJW-treated APP^swe^/PS1^dE9^mice. In line with previous studies, the control mice had a higher abundance and diversity of gut microbiota compared to the AD mice (Honarpisheh et al., 2020; Kim et al., 2020). Extending the results of 16SrRNA sequencing, we focused on the change in the intestinal microbiota at the phylum and genus level. Exposure to KXSJW, increased levels of *Bacteroidota* and *Firmicutes* at the phylum level. *Bacteroidota* are commonly found in the human gastrointestinal tract and form beneficial relationships in humans. *Bacteroidota* have a wide range of metabolic potential and produce SCFAs, including succinate, propionate, acetate and butyrate as their main end products, which further increases their effects on the host (Zafar and Saier, 2021). *Firmicutes* is the major phylum colonizing the healthy human colon, capable of fermenting fiber and thought to be associated with human systemic immunity (Jordan et al., 2023). At the genus level, KXSJW increased the abundance of *Muribaculaceae_unclassified* and *Lachnospiraceae_NK4A136_group* in the gut microbiota. *Muribaculaceae* is a Gram-negative anaerobic bacterium widely distributed in the mouse gut which specializes in the fermentation of polysaccharides with the ability to produce propionates, known as beneficial SCFAs, which are essential for human health (Lagkouvardos et al., 2019). Similarly, *Lachnospiraceae,* belonging to the *Firmicutes* phylum have the ability to derive polysaccharides from the diet and to synthesize SCFAs such as butyrate (Vacca et al., 2020). Overall, KXSJW may have a potential role in the intervention of AD through modulation of beneficial gut microbes.

SCFAs are metabolites produced by the gut microbiota which may have indirect biological effects on the brain, through maintaining the integrity of the gut and BBB, regulating the immune system and reducing neuroinflammation (Colombo et al., 2021; Frausto et al., 2021). Studies have reported that the gut barrier is compromised in AD because of its close contact with the gut microbiota (He et al., 2023; Pellegrini et al., 2023). This specific relationship between SFCAs and the brain led us to investigate the influence of KXSJW on gut flora metabolites in APP^swe^/PS1^dE9^ mice. The GC-MS results demonstrated significant changes in acetic acid, butyric acid and isovaleric acid content after KXSJW intervention. We suggest that this may be due to alteration in the composition of the gut flora in APP^swe^/PS1^dE9^ mice by KXSJW. Acetic acid can modulate microglial phagocytosis and regulate the homeostatic metabolic state (Erny et al., 2021). Butyric acid is considered a major energy source for intestinal epithelial cells. Additionally, it has been demonstrated to reduce microglia-mediated neuroinflammation (Sun et al., 2020). Furthermore, butyrate improves intestinal barrier function and enhances mucosal immunity (Fu et al., 2019). Our study confirmed that KXSJW treatment significantly increased expression of ZO-1 and occludin proteins; important components of intestinal tight junctions, in APP^swe^/PS1^dE9^ mice, tending to restore intestinal barrier function. Therefore, KXSJW may contribute to the maintenance of the intestinal barrier function in AD mice by interfering with the composition of the intestinal flora and their metabolites.

Although different studies have presented differing findings, it is increasingly believed that the regulation of gastrointestinal peptide secretion is influenced by the intestinal flora and their metabolites (Leeuwendaal et al., 2021; Hamamah et al., 2023). Our study shows that KXSJW significantly regulates the secretion of GHRP-Ghrelin and glucagon. Studies have suggested that various pathological changes associated with AD may be reduced by treatment with ghrelin or ghrelin receptor agonists (Jeon et al., 2019). The available evidence suggests that the effects of herbal compounds in KXSJW on GHRP-Ghrelin and glucagon may result from modulation of intestinal flora and the composition of SCFAs, e.g., butyric acid (Alhabeeb et al., 2021).

Our findings provide new insights into the potential role of KXSJW in the pathogenetic mechanisms of, and a possible therapeutic role in the treatment of, AD. However, limitations of our research must be acknowledged. This is a single center study of small numbers of animals, though in a well-established animal model of AD. Individuals diagnosed with AD frequently present with behavioural and psychological symptoms of dementia (BPSD). In experimental animal models, BPSD can be evaluated employing the water maze test (Murayama et al., 2021). In this study, the cognitive function of APP^swe^/PS1^dE9^ mice was evaluated using the MWM. Nevertheless, a systematic evaluation of BPSD was not undertaken, despite the observation of some instances of aggressive behavior in the mice during the 2-month period of feeding. Variable dose-response of KXSJW was seen, for example, on effects on microbial species, different SCFAs, serum GHRP-Gherelin and Glucagon release. Dose-response requires further elucidation. The complexity of the composition of TCM requires studies that take into account potential synergistic and/or inhibitory effects between drugs, in addition to the efficiency of absorption of the active ingredients of TCM. Larger studies including other AD models are required. Future studies, including the use of advanced analytical techniques such as metabolomics and pharmacokinetics are required to deepen our understanding of the bioavailability and pharmacological effects of TCM.

## 6 Conclusion

This study employed behavioral analysis, histopathology, and 16S rDNA sequencing to investigate the effectiveness of KXSJW in an animal model of AD.

KXSJW significantly intervened at multiple levels, reducing cognitive impairment and amyloid deposition in APP^swe^/PS1^dE9^ mice. Additionally, KXSJW alleviated the pathologic features of AD by modulating the gut microbiota and their metabolites. Specifically, KXSJW exhibited significantly modulated *Firmicutes* and its major metabolite, butyric acid. This may represent a potential therapeutic target for KXSJW, especially in maintaining intestinal barrier function and the secretion of related hormones.

This study confirms the effect of KXSJW on improving learning and cognition and modulating dysregulation of gut flora and their metabolites in APP^swe^/PS1^dE9^ mice and its potential to treat AD.

## Data Availability

The data presented in the study are deposited in the NCBI repository, accession number PRJNA1241812; available at https://www.ncbi.nlm.nih.gov/search/all/?term=PRJNA1241812.

## Abbreviations

AD, Alzheimer’s disease; EECs, enteroendocrine cells; GI, gastrointestinal; GC-MS, Gas chromatography-mass spectrometry; MWM, Morris Water Maze; SCFAs, short chain fatty acids; TCM, Traditional Chinese Medicine; BBB, blood-brain barrier; HE, Hematoxylin-Eosin; ELISA,Enzyme linked immunosorbent assay; OTUs, observed species operational taxonomic units; PCA, Principal Component Analysis; PCoA, Principal Coordinate Analysis Further detailed research on therapeutic mechanisms of KXSJW is required.
